# A Fungus-Specific Protein Domain Is Essential for RasA-Mediated Morphogenetic Signaling in *Aspergillus fumigatus*

**DOI:** 10.1128/mSphere.00234-16

**Published:** 2016-11-30

**Authors:** Qusai Al Abdallah, Tiffany S. Norton, Amy M. Hill, Lawrence L. LeClaire, Jarrod R. Fortwendel

**Affiliations:** a Department of Microbiology and Immunology, University of South Alabama, Mobile, Alabama, USA; b Department of Biochemistry and Molecular Biology, University of South Alabama, Mobile, Alabama, USA; c Department of Clinical Pharmacy, University of Tennessee Health Science Center, Memphis, Tennessee, USA; Carnegie Mellon University

**Keywords:** *Aspergillus*, Ras, actin, morphogenesis, signal transduction

## Abstract

Ras proteins function as conserved regulators of eukaryotic growth and differentiation and are essential signaling proteins orchestrating virulence in pathogenic fungi. Here, we report the identification of a novel N-terminal domain of the RasA protein in the filamentous fungus *Aspergillus fumigatus*. Whereas this domain is absent in Ras homologs of higher eukaryotes, the N-terminal extension is conserved among fungi and is characterized by a short string of two to eight amino acids terminating in an invariant arginine. For this reason, we have termed the RasA N-terminal domain the invariant arginine domain (IRD). Through mutational analyses, the IRD was found to be essential for polarized morphogenesis and asexual development, with the invariant arginine residue being most essential. Although IRD truncation resulted in a nonfunctional Ras phenotype, IRD mutation was not associated with mislocalization of the RasA protein or significant changes in steady-state RasA activity levels. Mutation of the RasA IRD diminished protein kinase A (PKA) activation and resulted in decreased interaction with the Rho-type GTPase, Cdc42. Taken together, our findings reveal novel, fungus-specific mechanisms for Ras protein function and signal transduction.

**IMPORTANCE** *Aspergillus fumigatus* is an important fungal pathogen against which limited treatments exist. During invasive disease, *A. fumigatus* hyphae grow in a highly polarized fashion, forming filaments that invade blood vessels and disseminate to distant sites. Once invasion and dissemination occur, mortality rates are high. We have previously shown that the Ras signaling pathway is an important regulator of the hyphal growth machinery supporting virulence in *A. fumigatus*. Here, we show that functional Ras signaling in *A. fumigatus* requires a novel, fungus-specific domain within the Ras protein. This domain is highly conserved among fungi, yet absent in higher eukaryotes, suggesting a potentially crucial difference in the regulation of Ras pathway activity between the human host and the fungal pathogen. Exploration of the mechanisms through which this domain regulates signaling could lead to novel antifungal therapies specifically targeting fungal Ras pathways.

## Abstract (precis)

*Aspergillus fumigatus* is an important fungal pathogen against which limited treatments exist. During invasive disease, *A. fumigatus* hyphae grow in a highly polarized fashion, forming filaments that invade blood vessels and disseminate to distant sites. Once invasion and dissemination occur, mortality rates are high. We have previously shown that the Ras signaling pathway is an important regulator of the hyphal growth machinery supporting virulence in *A. fumigatus*. Here, we show that functional Ras signaling in *A. fumigatus* requires a novel, fungus-specific domain within the Ras protein. This domain is highly conserved among fungi, yet absent in higher eukaryotes, suggesting a potentially crucial difference in the regulation of Ras pathway activity between the human host and the fungal pathogen. Exploration of the mechanisms through which this domain regulates signaling could lead to novel antifungal therapies specifically targeting fungal Ras pathways.

## INTRODUCTION

The Ras superfamily is comprised of membrane-associated proteins that display GTPase activities and function to orchestrate a variety of cellular processes. Because of their importance in cell development and differentiation, Ras proteins have been heavily studied in mammalian cells to gain insight into the process of tumor development (1). In human cells, multiple highly related Ras isoforms are encoded: H-ras, N-ras, and K-ras (K-ras4A and K-ras-4B). Although these proteins share a regulatory network of proteins for activation, they display differences in their posttranslational modification pathways and thus have distinct membrane localization, signaling output, and cellular functions (2–4). In fungi, Ras signaling has been extensively studied in the model yeast organism *Saccharomyces cerevisiae*. The *S. cerevisiae* genome encodes two Ras proteins, Ras1p and Ras2p. Both proteins control important cellular processes such as cell cycle progression, pseudohyphal growth, and stress response (5). Although Ras1p and Ras2p proteins share largely overlapping cellular functions, they display distinct expression patterns (5). Additionally, both proteins exhibit sequence similarity to human H-ras. Overexpression of human H-ras in yeast complements Ras2p deficiency, suggesting conservation of biological functions between human and yeast Ras proteins (6, 7).

Due to their multiple biological roles in the fungal cell, Ras proteins are important mediators of virulence in pathogenic fungi (8). In the human-pathogenic fungus *Aspergillus fumigatus*, two Ras proteins have been identified and characterized. RasA exhibits sequence similarity to human H-ras, whereas RasB is only found in filamentous fungi (9–11). Although each protein signals for specific biological functions, both proteins also play overlapping roles in fungal growth, development, and pathogenesis (8). The frequently encountered ability of Ras subfamily members to display overlapping functions is likely due to the highly conserved domain architecture that is common among Ras homologs. The conserved structure potentially allows for promiscuous effector interactions, especially when Ras proteins are overexpressed during genetic manipulations. Ras subfamily protein structure can be divided into a “G domain” and a hypervariable region (HVR). The G domain comprises 85 to 90% of Ras primary structure and contains the amino acid sequences required for guanine nucleotide binding, protein activation/inactivation cycles, and effector interactions. In contrast, the HVR is located at the C terminus of Ras proteins, where specific posttranslational modifications, including prenylation and palmitoylation, regulate protein localization to cell membranes.

Ras proteins have been examined as potential therapeutic targets against cancer and invasive fungal diseases in human and fungal cells, respectively. In the exploration for anti-Ras therapies directed against cancer cells, the proteins involved in posttranslational modifications of the HVR have been a major focus. Primary examples of work regarding HVR-directed compounds include (i) the development and therapeutic pursuit of compounds targeting prenylation (12), (ii) the identification of novel compounds that inhibit CAAX proteolysis (13), and (iii) the discovery of a small molecule that acts to disrupt the Ras palmitoylation cycle (14). The therapeutic goal of each of these agents is to alter Ras-membrane interactions and disrupt signal transduction. In contrast to the HVR, the G domain of Ras has proven to be a difficult target for novel drug development. The most enticing anti-Ras mechanism for targeting the G domain would result in blocking the Ras-GTP interaction and thereby inhibiting Ras protein activation and downstream signaling. However, due to the high-affinity Ras-GTP interaction, generation of a small molecule for successful inhibition of GTP binding is highly unlikely (15). The Ras G domain is also highly conserved among human and fungal Ras homologs, making this core structure difficult to explore for novel compounds with high specificity for the fungal pathogen. Thus, despite their potential, targeting of conserved aspects of Ras signaling will always carry the risk of impacting both the invading fungal pathogen and the human host. A better solution may be to explore fungus-specific domains that mediate Ras protein function and localization in pathogenic fungi. This approach carries the promise of identifying novel fungus-specific targets.

In the present study, we report the identification and characterization of a novel, fungus-specific domain of the *A. fumigatus* RasA protein. We show that this novel domain, located at the extreme N terminus, is conserved among the RasA homologs of numerous fungal organisms but is not found in the Ras proteins of higher eukaryotes. Mutation of this domain resulted in phenotypes indicative of a nonfunctional *A. fumigatus* Ras protein, including reduced hyphal growth, compact colony morphology, and delayed establishment of polarity. Despite these abnormalities, RasA protein localization and activation were unaffected. Our studies reveal that proper RasA signal transduction, at least via the Cdc42 and protein kinase A (PKA) pathways, is dependent on this fungus-specific domain in *A. fumigatus*. Since this novel domain is highly conserved among fungal RasA homologs, these findings suggest a potentially new mechanism for fungal regulation of Ras signaling output among fungal organisms in general.

## RESULTS

### Fungal Ras proteins contain a conserved N*-*terminal domain.

**FIG 1:**
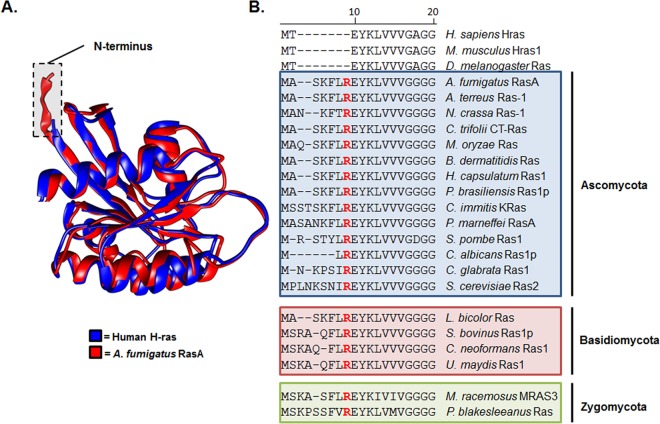
**T**he RasA protein displays an extended N terminus harboring an invariant arginine residue. (A) Comparison of Ras protein structure. A ribbon representation of H-ras (blue) (16) (PDB entry 4EFL) was superimposed with the predicted structure of RasA (red) using UCSF Chimera (54). Chimera was developed by the Resource for Biocomputing, Visualization, and Informatics at the University of California, San Francisco. Homology modeling of RasA structure was predicted using the known H-Ras structure as a template. The RasA amino acid sequence was mapped to the H-Ras structure using ProtSkin (55). The figure displays RasA amino acids 1 to 166. (B) Multiprotein alignment of N-terminal RasA homolog sequences from multiple fungal species and higher eukaryotes. Organisms and Ras homologs (with GenBank accession numbers in parentheses) employed for alignment include *Homo sapiens* H-ras (AAM12630.1), *Mus musculus* Hras1 (AAQ81319.1), *Drosophila melanogaster* Ras1 (AAF15514.1), *Aspergillus fumigatus* RasA (EAL91488.1), *Aspergillus terreus* Ras-1 (EAU31971.1), *Neurospora crassa* Ras-1 (P22126.1), *Colletotrichum trifolii* CT-Ras (AAC03781.1), *Magnaporthe* *oryzae* Ras (ELQ42826.1), *Blastomyces* *dermatitidis* Ras (EEQ88958.1), *Histoplasma* *capsulatum* Ras (EEH06649.1), *Paracoccidioides brasiliensis* Ras1p (AAZ81605.2), *Coccidioides immitis* K-Ras (KMU89545.1), *Penicillium marneffei* RasA (EEA28487.1), *Schizosaccharomyces pombe* Ras1 (CAB11218), *Candida albicans* Ras1p (AF177670.1), *Candida glabrata* Ras (XP_445167.1), *Saccharomyces cerevisiae* Ras2 (DAA10447.1), *Laccaria* *bicolor* Ras (AAD01987.1), *Suillus bovinus* Ras1p (AF250024.1), *Cryptococcus neoformans* Ras1 (AF294647.1), *Ustilago maydis* Ras1 (AAO19640.1), *Mucor racemosus* MRAS3 (AAA83379.1), and *Phycomyces* *blakesleeanus* Ras (OAD74690.1). The areas shaded in blue, red, and green denote organisms characterized as *Ascomycetes*, *Basidiomycetes*, or *Zygomycetes*, respectively. Note the invariant arginine residue (highlighted as red text) located in the same position for each fungal RasA homolog.

To delineate potential differences between fungal and human Ras proteins, we first compared predicted protein structure and amino acid composition of *A. fumigatus* RasA with the closest human homolog, H-ras. The crystal structure for H-ras has been solved previously (16) and was utilized to model the three-dimensional structure of the *A. fumigatus* RasA protein. Like the human H-ras protein, RasA was predicted to form a G domain composed of six β-pleated sheets and five α-helices (Fig. 1A), with an extended C-terminal hypervariable region (not shown). However, a major difference noted from the structure comparisons was the extended N terminus of the RasA protein (Fig. 1A). In *A. fumigatus*, this N-terminal region is located upstream of the highly conserved G domain and is comprised of 7 amino acids. By comparison, Ras homologs from higher eukaryotes, including *Homo sapiens* (H-ras), *Mus musculus* (Hras1), and *Drosophila melanogaster* (Ras) encode only 2 amino acids preceding the G-domain that include the initial methionine followed by a threonine residue (Fig. 1B). To examine the conservation of this N-terminal extension among divergent fungal species, we performed a multiprotein alignment using the RasA homologs from multiple fungal species (Fig. 1B). Protein sequences used for alignment consisted of the N-terminal portions of each RasA homolog, up to and including the highly conserved di-glycine repeat residues. These residues compose part of the G1 motif that directly binds the beta phosphate of GTP and GDP (17). The fungal N-terminal extension consisted of two to eight amino acids and was not found in the Ras homologs of higher eukaryotes, including human H-ras (Fig. 1B). This extended N-terminal domain contained an invariant arginine residue in the same position within each fungal RasA homolog, just upstream of the highly conserved G1 domain (Fig. 1B). Therefore, we have termed this fungus-specific domain the invariant arginine domain (IRD). Although the invariant arginine residue was positioned in an identical location for all fungal RasA homologs, the residues upstream of the invariant arginine displayed high conservation only among closely related fungi and increased variability among divergent species.

### The RasA IRD is required for hyphal growth and morphogenesis.

**FIG 2:**
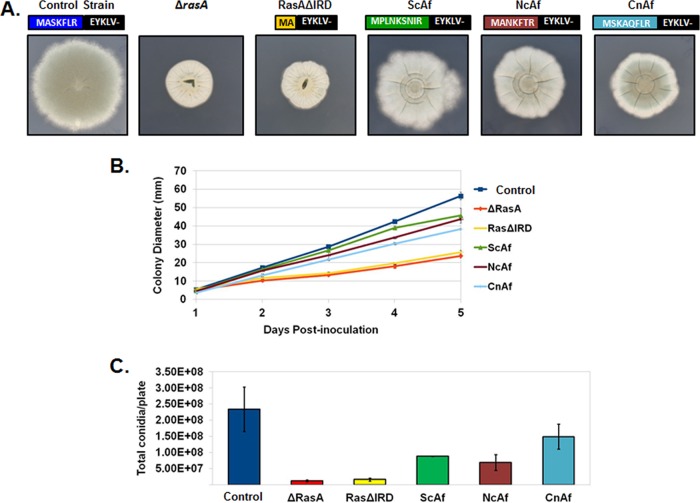
The IRD is required for hyphal growth and asexual development. (A) Colony morphologies of the control strain, the *rasA* deletion mutant (Δ*rasA*), the IRD truncation mutant (RasAΔIRD), and mutants expressing N-terminal chimeras including the IRD regions from *S. cerevisiae* (ScAf), *N. crassa* (NcAf), and *C. neoformans* (CnAf). For each mutant, the N-terminal RasA protein sequence resulting from mutational analysis is displayed. Equal numbers of conidia (10^4^ total conidia) were inoculated onto the middle of AMM agar plates and allowed to grow for 5 days at 37°C. (B) Graphical representation of colony diameters from each strain (10^4^ total conidia) over 5 days of growth at 37°C. Each culture was assayed in triplicate, and results were averaged. The graph displays the average for each time point ± standard deviation. (C) Enumeration of conidia produced by each strain after 3 days of growth at 37°C. Equal numbers of conidia (2 × 10^4^ total conidia) were spread onto AMM agar and incubated for 3 days. Conidia were harvested from each plate in 20 ml of water, washed 3 times with water, resuspended in 10 ml of water, and enumerated. Data are presented as the total conidia per plate and are the average from two experiments ± standard deviation.

In order to determine the importance of the RasA IRD, we first generated a truncation mutant lacking the entire IRD, deleting the DNA sequence encoding amino acid positions 3 through 7 (Fig. 2A). The truncated cDNA was cloned under control of the endogenous *rasA* promoter using vector pAGRP and introduced into the Δ*rasA* mutant, as previously described (18). Expression of the IRD truncation mutant from the pAGRP vector also introduces an N-terminal green fluorescent protein (GFP) tag, allowing subsequent localization studies. Protein expression was confirmed by Western blot analysis (see Fig. S1 in the supplemental material). This novel RasA mutant was named RasAΔIRD. Colony morphology, radial growth, and asexual reproduction were scored for RasAΔIRD and compared to those of control strains. The strains used as controls included the Δ*rasA* mutant (9), to account for phenotypes exhibited by loss of RasA, and the previously generated GFP-RasA strain (18). The GFP-RasA strain was used here a positive growth control because the genetic manipulations involved in generation of the GFP-RasA strain are identical to those in the RasAΔIRD mutant described herein (i.e., both strains employ the pAGRP vector for integration and expression of a GFP-fused Ras protein in the Δr*asA* genetic background). This strain will hereafter be referred to as the “control strain.” The RasAΔIRD mutant formed slow-growing, compact colonies and conidiated poorly on minimal media, similar to the Δ*rasA* mutant (Fig. 2A to C). To ensure GFP tagging of the RasAΔIRD mutant was not underlying the observed growth defect, we also generated a mutant lacking the GFP fusion. This IRD truncation mutant was expressed using the same pAGRP vector after removal of the GFP coding sequence and showed a similar growth defect when compared to the GFP-tagged version (data not shown). The GFP-fused RasAΔIRD strain was utilized for the remainder of this study to facilitate localization and protein interaction studies. These data suggested that the fungus-specific RasA N terminus was essential for RasA function in *A. fumigatus*.

10.1128/mSphere.00234-16.1 Figure S1  Expression levels of chimeric RasA proteins. (A) Expression levels were compared by quantitative Western blotting using an anti-GFP antibody. Loading controls were performed to ensure that equal amounts of lysate were loaded for Western blot analysis. ScAf, CnAf, and NcAf are strains expressing the N-terminal regions from *S. cerevisiae* Ras2, *C. neoformans* Ras1, and *N. crassa* Ras-1, respectively, fused to the *A. fumigatus* RasA G domain. A negative control for the anti-GFP antibody included lysate from the Δ*rasA* mutant and is shown in Fig. 2. (B) The quantification of the Western blot bands was performed using ImageJ software. Expression levels were obtained by computing the expression level ratios of RasA chimeras to RasA from the control strain. The graph represents the average result from three separate experiments ± standard deviation. Download Figure S1, JPG file, 0.7 MB. Copyright © 2016 Al Abdallah et al. 2016 Al Abdallah et al. This content is distributed under the terms of the Creative Commons Attribution 4.0 International license.

10.1128/mSphere.00234-16.2 Figure S2  Expression levels of alanine-scanning mutants of RasA. (A) Expression levels were compared by quantitative Western blotting using an anti-GFP antibody. Loading controls were performed to ensure that equal amounts of lysate were loaded for Western blot analysis. (B) The quantification of Western blot bands was performed using ImageJ software. Expression levels were obtained by computing the expression level ratios of RasA alanine-scanning mutants to RasA from the control strain. The graph represents the average result from three separate experiments ± standard deviation. Download Figure S2, TIF file, 0.6 MB. Copyright © 2016 Al Abdallah et al. 2016 Al Abdallah et al. This content is distributed under the terms of the Creative Commons Attribution 4.0 International license.

To analyze functional conservation of the RasA IRD, chimeric RasA constructs were generated by fusing the IRD regions from the RasA orthologs of three separate fungal organisms to the G domain of the *A. fumigatus* RasA protein. The IRD regions from *Saccharomyces cerevisiae*, *Neurospora crassa*, and *Cryptococcus neoformans*, each varying in composition and length (Fig. 1B), were used to replace the *A. fumigatus* IRD followed by expression in the Δ*rasA* mutant background, as described above (Fig. 2A). Colony morphology, growth rate, and conidiation were again analyzed during growth on minimal medium and compared to those of controls. Interestingly, each fungal IRD was able to partially complement the deletion of the *A. fumigatus* IRD, but none were able to completely restore wild-type growth and morphology (Fig. 2B). Expression of the *C. neoformans* IRD chimera (CnAf) was associated with the lowest recovery of radial growth rate but the highest recovery of conidiation (Fig. 2C). Note that for each of the IRD chimeras, the invariant arginine residue remained intact. These findings suggested that the specific amino acid composition of the *A. fumigatus* IRD was required for RasA function during hyphal growth and morphogenesis.

**FIG 3:**
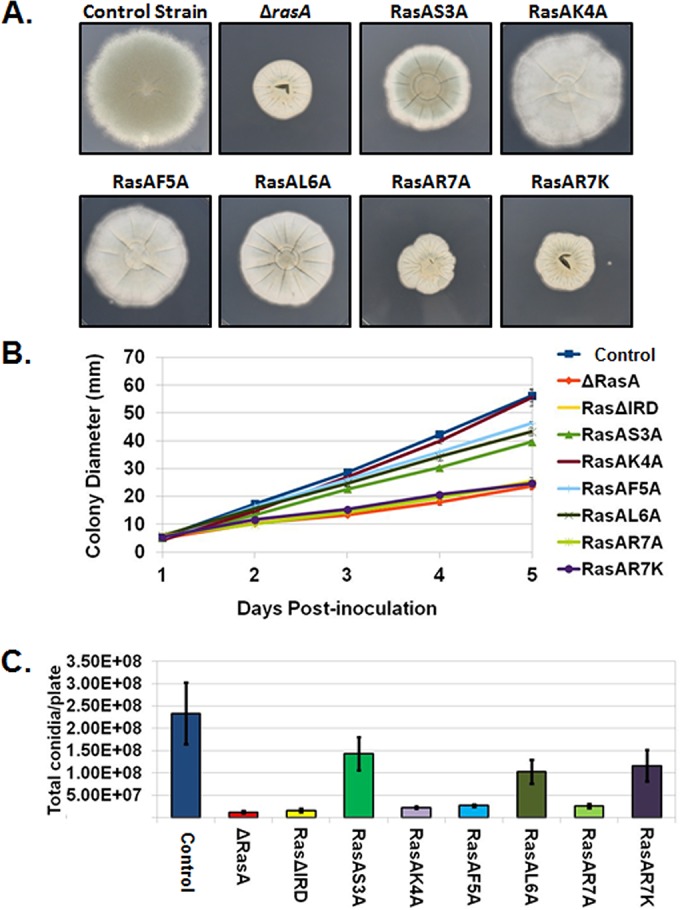
The invariant arginine residue is critical for IRD-mediated RasA functions. (A) Colony morphologies of the control strain, the *rasA* deletion mutant (Δ*rasA*), the alanine-scanning mutants (RasAS3A, RasAK4A, RasAF5A, RasAL6A, and RasAR7A), and the mutant with lysine mutation of the invariant arginine residue (RasAR7K). Equal numbers of conidia (10^4^ total conidia) were inoculated onto the middle of AMM agar plates and allowed to grow for 5 days at 37°C. (B) Graphical representation of colony diameters from each strain (10^4^ total conidia) over 5 days of growth at 37°C. Each culture was assayed in triplicate, and results were averaged. The graph displays the average for each time point ± standard deviation. (C) Enumeration of conidia produced by each strain after 3 days of growth at 37°C. Equal numbers of conidia (2 × 10^4^ total conidia) were spread onto AMM agar and incubated for 3 days. Conidia were harvested from each plate in 20 ml of water, washed 3 times with water, resuspended in 10 ml of water, and enumerated. Data are presented as the total conidia per plate and are the average from two experiments ± standard deviation.

To further investigate the importance of the *A. fumigatus* RasA N terminus, an alanine-scanning approach was employed to mutate each amino acid position within the RasA IRD. RasA cDNAs, mutated to encode the desired mutations, were cloned under control of the RasA promoter and expressed in the Δ*rasA* background, as previously described (18). Similar to expression of the N-terminal chimeras, alanine substitutions of serine, lysine, phenylalanine, and leucine only partially compensated for the loss-of-growth phenotype (Fig. 3A and B). With the exception of the RasAK4A mutant, all alanine mutants displayed slower radial growth rates than the control strain (Fig. 3A and B). Additionally, the RasAR7A mutant developed a compact colony morphology similar to that of the Δ*rasA* strain, suggesting the invariant arginine residue to be the most critical of the IRD (Fig. 3A and B). To determine if a more conservative amino acid substitution for the invariant arginine would be better able to support growth, an arginine-to-lysine substitution (RasAR7K) was generated. Interestingly, the RasAR7K mutant also displayed poor radial growth and compact colony morphology similar to those of the RasAR7A and Δ*rasA* mutants (Fig. 3A and B). In addition, conidiation was decreased in all mutants, with the RasAR7A mutant displaying a phenotype similar to those of the Δ*rasA* and RasAΔIRD mutants (Fig. 3C).

**FIG 4:**
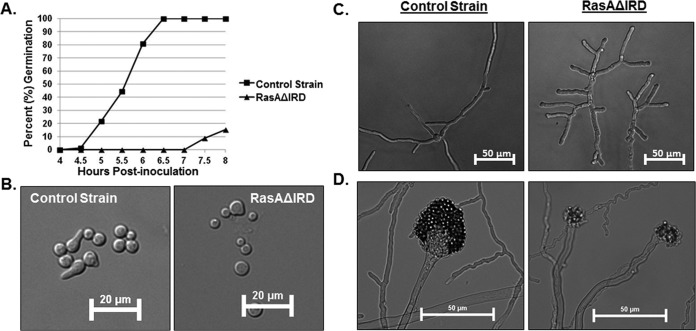
IRD truncation abrogates RasA function. (A) Comparison of germination rates between the control strain and the RasAΔIRD mutant. Equal numbers of conidia were inoculated onto AMM broth and incubated at 37°C. At the noted time points, samples were removed, and 100 random conidia from each strain were scored for germ tube production (polarity establishment). Data are presented as the percentage of germinated conidia for each time point. (B) Representative micrographs of germinating conidia from each strain after 5 h of incubation at 37°C. Note the irregular conidial size of the RasAΔIRD mutant, indicating higher variability in the timing of germination initiation. (C) Representative micrographs of hyphal development aberrancies in the RasAΔIRD mutant after 16 h of growth at 37°C. Conidia from each strain were cultured as described for the germination studies. Note the stunted, highly branched hyphae produced by RasAΔIRD. (D) Representative micrographs of conidiophore development in the control strain and RasAΔIRD mutant. After 24 h of culture at 37°C on AMM agar, the control strain produced conidiophores with swollen vesicles and numerous conidia arranged in chains (left panel). The RasAΔIRD mutant produced no conidiophores at 24 h of identical culture (data not shown) and produced distorted conidiophore structures by 48 h (right panel), characterized by minimally swollen vesicles and few conidia.

To more clearly define phenotypes caused by IRD mutation, the RasAΔIRD mutant was further characterized for germination rate, hyphal morphology, and conidiophore development. Similar to earlier results in a strain lacking RasA (9), the RasAΔIRD mutant displayed delayed establishment of polarity (Fig. 4A and B). Mature hyphal growth in the RasAΔIRD mutant was characterized by highly branched, dysmorphic hyphae with blunted tips (Fig. 4C). In agreement with the lack of conidiation of the RasAΔIRD strain noted previously, conidiophore development was also delayed and resulted in minimally swollen vesicles and misshapen phialides (Fig. 4D). Together, these phenotypes suggest that IRD truncation results in a largely nonfunctional RasA protein.

### Localization and activation of RasA are not affected by IRD truncation.

**FIG 5:**
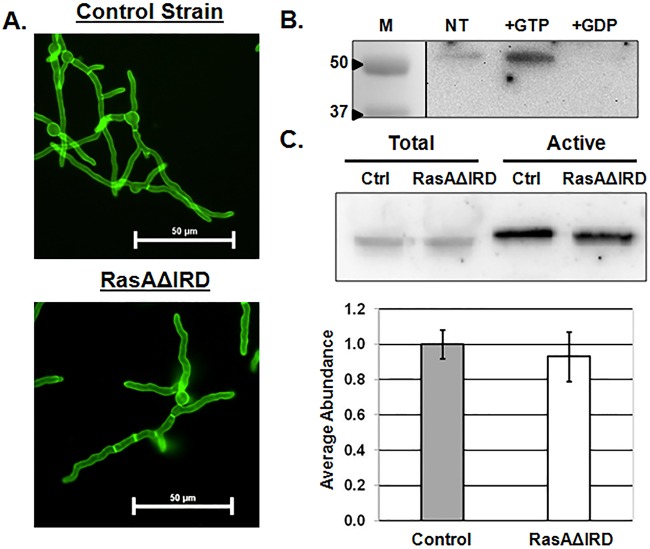
RasA localization and activation are not affected by IRD truncation. (A) Representative fluorescence micrographs of the control strain and RasAΔIRD. AMM broth cultures of each strain were incubated at 37°C until approximately equal hyphal lengths were achieved for each strain. Cultures were then mounted for fluorescence microscopy, and images were acquired using identical exposure settings for both strains. (B) Ras activation assay performed on lysate from the control strain comparing the amount of total Ras bound under no treatment (NT), after GTP preloading (+GTP), and after GDP preloading (+GDP). GFP-fused RasA is detected at 51 kDa using an anti-Ras antibody. The control assay was repeated twice to ensure the ability of RasA to bind the Raf1 RBD-agarose bead and to appropriately respond to activation (+GTP) and inactivation (+GDP). Lane M, molecular mass (kilodaltons) marker lane.(C, top panel) Western blot analysis of total RasA detected in lysates from each strain (Total) and the amount of active RasA detected by Raf1-RBD pulldown (Active). The gel image is representative of three independent experiments. Ctrl, control. (C, bottom panel) Quantification of active/total Ras ratios. Western blot bands were quantified by densitometry analysis, and the average signal abundance ratio of detectable active RasA to total RasA was calculated. Data are presented as an average from three replicates ± standard deviation. No statistical difference was noted in Ras activation levels between the control and RasAΔIRD mutant.

Plasma membrane localization and controlled activation of RasA are necessary for efficient signal transduction supporting hyphal morphogenesis, cell wall formation, and virulence (9, 18, 19). Because IRD truncation resulted in phenotypes indicative of a nonfunctional Ras signaling network, we hypothesized that IRD mutations may alter either protein localization or activation. To examine effects of IRD mutation on RasA plasma membrane association, we analyzed the localization of the IRD truncation mutant, taking advantage of the N-terminal GFP tag added by the pAGRP expression vector (18). *Aspergillus* minimal medium (AMM) broth cultures were incubated until equivalent lengths of hyphae were developed for the control strain (12 h, 37°C) and the RasAΔIRD mutant (16 h, 37°C). At this stage of hyphal development, RasA localization in the control strain was primarily confined to the plasma membrane, with even distribution along the hyphal perimeter and strong fluorescence at septa (Fig. 5A). These results were in agreement with earlier work employing this strain (18). Localization of the RasAΔIRD protein was identical to that of the wild-type RasA protein in the control strain (Fig. 5A). These data indicate that the fungus-specific RasA N terminus is not required for protein localization to the plasma membrane.

To examine the possibility that IRD mutation negatively affects RasA activity levels, a commercially available Ras activation assay was employed. The Ras activation assay allows selective pulldown of active, GTP-bound forms of Ras by utilizing agarose beads coated with the Raf-1 Ras-binding domain (RBD) (20). Adaptation of this assay to *A. fumigatus* showed that active RasA was detectable in control lysates (Fig. 5B), indicating the ability of GTP-bound RasA to associate with the Raf-1 RBD provided in the Ras activation assay. Additionally, the amount of active protein detected was increased when lysates were preloaded with GTP and was decreased when lysates were preloaded with GDP (Fig. 5B). Thus, the Ras activation assay is able to detect changes in RasA activation state. To test the effects of IRD mutation, Raf-1 RBD-bound active RasA and total protein fractions from the control and RasAΔIRD strains were immunoblotted side by side (Fig. 5C). The ratio of active RasA versus total RasA was used as a measure of steady-state activation levels. Statistical analysis of densitometric data from assays run in biological triplicate confirmed no changes in steady-state RasA activation levels between the control and RasAΔIRD strains (Fig. 5C). Taken together, our data suggest that IRD mutation does not affect RasA localization or activation.

### The IRD is required for optimal signal transduction in *A. fumigatus.*

Because RasA localization and activation appeared normal in the RasAΔIRD mutant, we next hypothesized that truncation of the IRD may alter the ability of RasA to interact with and activate its downstream signaling partners. Although no direct RasA binding partners have been identified in *A. fumigatus*, common downstream components of Ras signaling in fungi include the protein kinase A (PKA) pathway, via activation of adenylate cyclase, and the Rho-type GTPase, Cdc42, typically through interaction with the Cdc42 guanine nucleotide exchange factor (GEF) protein, Cdc24 (8). In yeast and filamentous fungi alike, these pathways are important regulators of growth, morphogenesis, and stress response.

**FIG 6:**
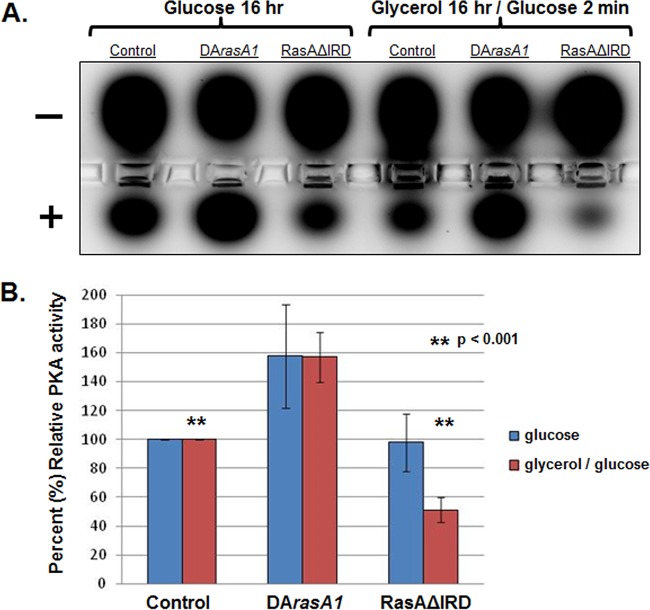
PKA activation is deficient in the RasAΔIRD mutant. (A) PKA activity assay performed on lysates of the control strain, a strain expressing constitutively active RasA (DA*rasA1*), and the RasAΔIRD mutant. Equal numbers of conidia from each strain were cultured in either (i) AMM glucose-based medium (Glucose 16 hr) overnight or (ii) AMM modified to contain glycerol as the sole carbon source, followed by a 2-min stimulation with glucose (Glycerol 16 hr/Glucose 2 min). Lysates were generated from each strain and condition and assayed for PKA activity using the PepTag nonradioactive cAMP-dependent protein kinase assay (Promega). The agarose gel electrophoresis image is representative of three independent experiments and shows migration of phosphorylated PKA-target peptide toward the cathode (+) and unphosphorylated target toward the anode (−). Control experiments, including addition of the bovine PKA catalytic subunit and exogenous cAMP to measure induction of PKA activity, are provided in Fig. S3 in the supplemental material. (B) Densitometric quantification of phosphorylated PKA-target peptide (migrating toward the cathode) for each strain, relative to the control strain. Results are the average from three separate experiments ± standard deviation. Data were compared using Student’s *t* test.

To test for alterations in PKA activity levels due to RasA IRD deletion, we utilized a commercially available assay that measures relative amounts of phosphorylated PKA substrate. This assay has been utilized in multiple fungal species to assay PKA activity and relies on differential migration of a conserved PKA substrate peptide in an agarose gel based on phosphorylation status (21, 22). In yeast, PKA pathway activity is responsive to the available carbon source, with relatively high activity levels being induced in glucose-based media compared with lower activity levels in glycerol-based media (23). Because a role for RasA in activation of PKA has not been directly tested in *A. fumigatus*, we first cultured strains expressing wild-type RasA, constitutively active RasA, or the RasAΔIRD mutant in glucose-based minimal media for 16 h and assayed steady-state PKA activity levels. The constitutively active RasA mutant used here (DA*rasA1*) expresses a single copy of activated RasA in the Δ*rasA* genetic background (19). After 16 h of culture, hyphae from each strain were collected, lysates were extracted, and the PKA assay was completed per the manufacturer’s protocols. The *A. fumigatus* strain expressing wild-type RasA displayed a strong active PKA signal, as indicated by migration of a proportion of the PKA substrate toward the cathode (Fig. 6A). By comparison, the DA*rasA1* mutant displayed an increase in active PKA signal, indicating RasA activation results in increased PKA activity in *A. fumigatus* (Fig. 6A and B). Although the RasAΔIRD mutant displayed greater variation in PKA activity readout, a statistically significant difference in steady-state PKA activity levels was not detected (Fig. 6A and B). To test for alterations in PKA activation levels upon carbon source stimulation, each of the strains was cultured overnight in glycerol-based minimal medium, followed by addition of 2% glucose (final concentration) for 2 min. This protocol is in agreement with yeast-based assays for stimulation of PKA pathway activity (24). Upon glucose addition, strains expressing wild-type and activated RasA displayed a similar pattern to before, with the DA*rasA1* mutant producing relatively high levels of PKA activation (Fig. 6A and B). In contrast, the RasAΔIRD mutant was consistently unable to produce PKA activity levels equivalent to those of the wild-type RasA strain but instead displayed low levels of PKA activity (Fig. 6A and B). These data indicate that the RasA IRD is required for PKA pathway activation in response to glucose stimulation.

10.1128/mSphere.00234-16.3 Figure S3  Control reactions for the PKA activity assay. (A) Negative (NC) and positive (PC) control reactions were run using the kit components following the manufacturer’s protocols. Reaction conditions were identical, except for the addition of the bovine PKA catalytic subunit to the PCR. (B) Activation of PKA activity in *A. fumigatus* lysate via addition of exogenous cAMP. Lysates from the control strain were isolated and quantified as described for Fig. 6. For cAMP stimulation, 50 µg of total protein lysate was then treated with 1 µM cAMP (Sigma) at room temperature for 30 min. NT, no treatment. Download Figure S3, JPG file, 0.5 MB. Copyright © 2016 Al Abdallah et al. 2016 Al Abdallah et al. This content is distributed under the terms of the Creative Commons Attribution 4.0 International license.

**FIG 7:**
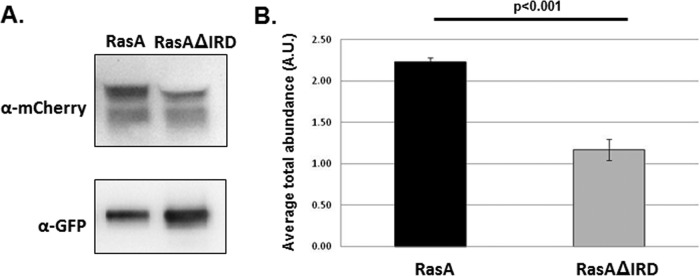
RasA interaction with Cdc42 is mediated by the IRD in *A. fumigatus*. (A) Lysates of the control strain and RasAΔIRD mutant were immunoprecipitated with anti-GFP agarose beads and subjected to Western blot analysis with either an anti-mCherry antibody (for mCherry-Cdc42 detection) or an anti-GFP antibody (for Ras protein detection). Images are from one representative experiment. Control experiments, including the inverse-pulldown reaction, are provided in Fig. S4 in the supplemental material. (B) Quantification of the amount of mCherry-Cdc42 immunoprecipitated with either RasA (from the control strain) or RasAIRD. Densitometry analysis was performed using ImageJ software, and the ratio of anti-mCherry signal to anti-GFP signal was calculated. The graph represents the average result from three separate experiments ± standard deviation. Data were compared using Student’s *t* test.

In the yeast organisms *S. cerevisiae* and *C. neoformans*, Ras homologs interact with and activate Cdc42 via Cdc42-GEF, and Cdc24 (25, 26). The outcome of this interaction is to regulate complex morphogenetic networks. To see if RasA-Cdc42 interactions are also altered in the IRD truncation mutant, we utilized a coimmunoprecipitation assay, as previously described (27). Strains coexpressing a single copy of either GFP-RasA or GFP-RasAΔIRD along with mCherry-Cdc42 in a wild-type *A. fumigatus* genetic background were generated. Each strain was cultured overnight in glucose minimal medium (GMM), and coimmunoprecipitation of whole-cell lysates was performed using agarose beads coated with anti-GFP antibodies (Chromotek). Western blots of each precipitation reaction were probed with either a polyclonal anti-GFP antibody to detect the amount of RasA or RasAΔIRD protein precipitated or with a monoclonal anti-mCherry antibody to detect Cdc42 (Fig. 7A). Densitometric analyses were performed to express the relative amount of precipitated protein as a ratio of Cdc42 to RasA or Cdc42 to RasAΔIRD. Under the conditions tested, immunoprecipitations of the RasAΔIRD mutant contained significantly less Cdc42 than those from a strain expressing the wild-type RasA protein (Fig. 7B). These data suggest that, in addition to the PKA pathway assayed above, the RasA IRD is also required for proper morphogenetic signaling though Cdc42 pathways in *A. fumigatus*.

10.1128/mSphere.00234-16.4 Figure S4  Control reactions for coimmunoprecipitation of RasA and Cdc42. (A) Coomassie-stained SDS-PAGE of the input (I), unbound (U), and bound (B) fractions from both the GFP-Ras/mCherry-Cdc42- and GFP-RasIRD/mCherry-Cdc42-expressing strains. The left panel shows results from coimmunoprecipitation reactions using the GFP-Trap agarose beads, and the right panel shows results from reactions with the RFP-Trap agarose beads (Chromotek). MW, molecular weight marker. (B) Western blot analysis of reverse coimmunoprecipitation reaction using the RFP-Trap agarose beads to immunoprecipitate mCherry-Cdc42 from each strain. The assay was run as described for GFP-Trap immunoprecipitation in the legend to Fig. 7. Download Figure S4, JPG file, 0.4 MB. Copyright © 2016 Al Abdallah et al. 2016 Al Abdallah et al. This content is distributed under the terms of the Creative Commons Attribution 4.0 International license.

### IRD mutation results in altered actin cytoskeleton dynamics and endocytosis.

**FIG 8:**
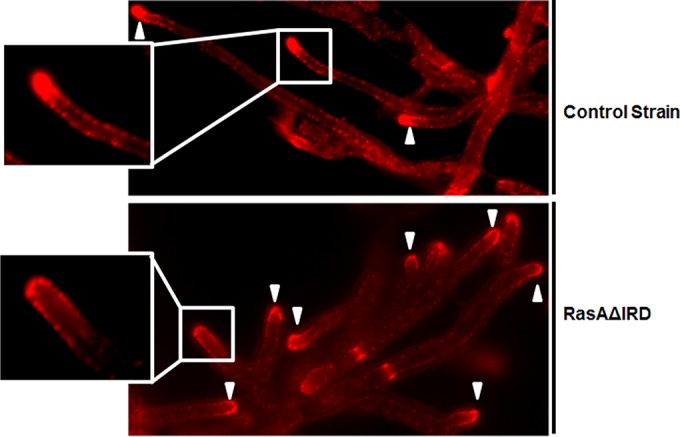
The IRD is required for RasA-mediated actin polarization. Immunofluorescence micrographs showing actin cytoskeleton distribution in the control strain (upper panel) and the RasAΔIRD mutant (lower panel). AMM broth cultures of each strain were incubated at 37°C until approximately equal hyphal lengths were achieved for each strain. Images were acquired under identical exposure times and are representative of the entire culture for both strains. Note the decreased presence of actin polarization to the hyphal tip in the RasAΔIRD mutant (inset panels). Arrowheads denote locations of hyphal tips for both panels.

In fungi, Ras network signaling via Cdc42 mediates actin cytoskeleton polarization (28, 29). Therefore, we hypothesized that the altered signal transduction of the RasAΔIRD mutant would also be associated with aberrancies in the actin cytoskeleton. To detect changes in the actin cytoskeleton, mature hyphae from the RasAΔIRD and control strains were formalin fixed and immunostained with an antiactin polyclonal antibody followed by a tetramethyl rhodamine isothiocyanate (TRITC)-labeled secondary antibody. As previously published, the wild-type *A. fumigatus* strain displayed condensed actin structures at the hyphal apex and clearly defined cortical actin patches along subapical compartments (Fig. 8). In contrast, the RasAΔIRD mutant displayed diminished localization of actin to hyphal tips, which coincided with decreased staining intensity of cortical actin patches in subapical compartments (Fig. 8).

**FIG 9:**
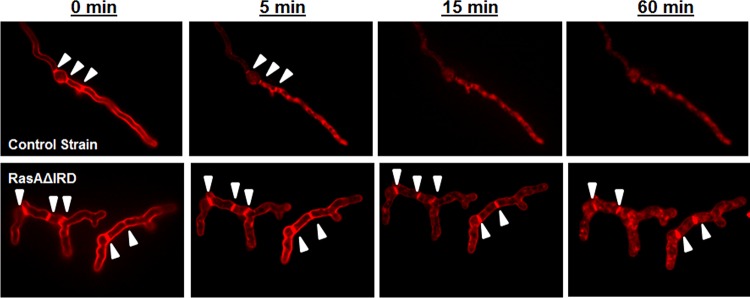
The endocytic rate is decreased in the RasAΔIRD mutant. Fluorescence micrographs showing FM4-64 uptake in a single hypha of the control strain (upper panels) and the RasAΔIRD mutant (lower panels) over 60 min at 37°C. AMM broth cultures of each strain were incubated at 37°C until approximately equal hyphal lengths were achieved for both strains. Cultures were then washed, loaded with 10 µM FM4-64 at 25°C, and further incubated at 37°C to monitor endocytic uptake of the dye. Images were acquired under identical exposure times. Note the delayed internal staining of RasAΔIRD hyphae (lower panels). Arrowheads denote locations of septa for both panels.

Alterations in actin localization to the hyphal tip and cortical actin patches are associated with a defective endocytosis rate in fungi (30). To examine endocytic uptake, the RasAΔIRD and control strains were cultured as described previously for RasA protein localization analysis and then preloaded with the endocytic marker FM4-64. FM4-64 is an amphiphilic styryl dye that, when preloaded onto fungal hyphae at 4°C, inserts into the outer leaflet of the plasma membrane (31). Subsequent uptake does not occur by passive diffusion, requiring active endocytic uptake instead (31). Dye uptake was followed over the course of 1 h at 37°C. Preloading of dye resulted in uniform labeling of the plasma membrane and septa at time zero (Fig. 9). Within 5 min of incubation at 37°C, the wild-type strain had internalized a significant portion of the FM4-64, as indicated by the irregular staining of the hyphae and loss of clear plasma membrane staining (Fig. 9). Dye uptake was nearly complete in the wild-type strain after only 15 min of incubation, with complete loss of plasma membrane staining (Fig. 9). Following an identical staining and endocytic uptake timeline, the RasAΔIRD mutant displayed prolonged staining of the plasma membrane at the hyphal perimeter and at septa, which was still present after 1 h of incubation (Fig. 9). Taken together, these data suggest that the RasA N terminus is required for RasA-mediated orchestration of actin organization and endocytic uptake in *A. fumigatus* to support polarized morphogenesis.

## DISCUSSION

Ras signaling networks are important pathways for probing fungal virulence mechanisms related to morphogenesis, metabolism, and stress response. The conservation of Ras protein structure between the human host and fungal pathogen provides a solid foundation for delineating similarities in function but can also hamper the development of anti-Ras therapies with fungal specificity. To successfully target this eukaryotic pathogen, more work focused on delineating differences between fungal and human Ras protein functions is needed. Our findings show that a fungus-specific, N-terminal extension of *A. fumigatus* RasA is required for mediation of signal output. Mutation of this N-terminal domain revealed that each amino acid residue within the N terminus at least partially contributes to RasA-orchestrated hyphal growth and asexual reproduction. In addition, truncation of the RasA N terminus caused delayed germination and altered hyphal morphology indicative of a nonfunctional RasA protein. This is the first report to identify fungus-specific aspects of Ras signaling in *A. fumigatus*. Importantly, this study focused on understanding the functional importance of a Ras protein domain that is conserved among numerous fungi but is not found in higher eukaryotes.

Mislocalization of Ras homologs from the plasma membrane has been shown to disrupt Ras signaling in a number of fungal species, including *A. fumigatus* (18, 32, 33). Ras protein mislocalization is typically driven by inhibition of lipid-based, posttranslational modifications that occur at the carboxy terminus. These include prenylation of the conserved cysteine within the CAAX motif, as well as palmitoylation of one or more conserved cysteines within the HVR (34). When these processes are inhibited, via either pharmacological or genetic means, Ras is misplaced from the plasma membrane, and specific elements of Ras signaling are disrupted due to loss of signaling context and/or activation (35). Regarding IRD truncation, we found RasA localization to be largely unaffected, implying that mislocalization is not a major reason for the noted loss of function. This finding is, perhaps, unsurprising due to the lack of proximity of the IRD to the HVR.

As a protein harboring intrinsic GTPase activity, Ras binds to and hydrolyzes GTP molecules to toggle between active and inactive confirmations. Active Ras is GTP bound, whereas inactive Ras is GDP bound. Ras-GTP/GDP binding requires five highly conserved regions called G motifs that collectively form the core structure of the G domain. Aiding in the cycle of GTP/GDP hydrolysis and exchange are GTPase-activating proteins (GAPs) and guanine nucleotide exchange factors (GEFs), respectively. GAPs associate with Ras-GTP and catalyze the relatively slow intrinsic rate of GTP hydrolysis of Ras, thereby acting as an inactivator (36). In contrast, GEFs bind to Ras-GDP and aid in the release of the GDP molecule so Ras can again bind the more readily available GTP molecule, thereby activating the Ras protein (36). The endpoint of the GAP/GEF interaction with Ras is to affect the steady-state levels of active Ras within the cell and alter the overall amount of Ras network activity. Therefore, steady-state levels of active Ras protein can be expected to change based on (i) inhibition of the intrinsic GTPase activity of the Ras protein, (ii) inhibition of the ability of Ras to bind GTP/GDP molecules, or (iii) alteration of Ras-GAP/GEF interactions. However, in our studies with the IRD truncation mutant, active RasA levels were not significantly impacted, indicating that these important Ras activation mechanisms are largely unaffected. Again, this result might be predicted based on the fact that the IRD resides outside of the conserved domains known to be required for Ras protein activation.

Because both localization and activation of RasA appeared normal in the IRD truncation mutant, we speculated that the IRD may instead play an important role in Ras signaling to downstream effectors. Indeed, we found that activation of PKA in response to glucose stimulation and RasA interaction with Cdc42 were both negatively impacted by IRD truncation. We also noted decreased actin polarization to hyphal tips and altered endocytic uptake in the mutant. These important cellular processes are known to be impacted by Cdc42 and PKA signaling in fungi and are likely underlying the defective growth patterns imparted by IRD truncation in *A. fumigatus* (37–40). It will be of interest to further pursue the spatiotemporal accumulation and organization of F-actin structures at the hyphal tip during early growth processes in the IRD mutant to see how the kinetics of RasA signaling affect this essential process. Our findings support several hypotheses that will be the focus of future work to understand fungus-specific aspects of signal transduction in pathogenic fungi.

The first hypothesis is that the IRD may be a site for posttranslational modification(s) modulating RasA signal output. Because the arginine residue is invariant among all fungal species examined, it is reasonable to speculate that the arginine residue may be an important methylation target. Non-histone protein methylation has recently emerged as a major regulator of cellular signaling in eukaryotes, affecting protein activation and protein-protein interactions (41). Arginine methylation of a Ras protein remains uncharacterized and, if true for fungal Ras proteins, would certainly be a departure from what is understood about the regulation of Ras network activity in human cells. We have, as of yet, been unable to detect the IRD peptide using standard trypsin digestion and mass spectrometry techniques (data not shown), making methyl-arginine detection by this method difficult. However, further studies are planned to test this exciting possibility.

A second hypothesis is that the IRD could function as a binding site for known and/or uncharacterized Ras effectors. For example, *S. cerevisiae* Ras2 is known to directly interact with adenylate cyclase to modulate cyclic AMP (cAMP) levels for protein kinase A (PKA) activation (42). In *Schizosaccharomyces pombe*, Ras1 directly interacts with the RhoGEF, Scd1, to activate Cdc42 (43, 44). Because we found alteration in both PKA and Cdc42 pathways upon IRD truncation, these specific interactions in *A. fumigatus* may require the IRD. Related to this hypothesis, the IRD might interact with scaffolding proteins important for efficient signaling through various networks (45, 46). Alternatively, the IRD may be directly involved in binding to entirely novel proteins that play important roles in fungal Ras signaling fidelity. Unbiased proteomics analyses and direct, protein-protein interaction experiments are being undertaken to identify potentially novel Ras interactions dependent on the IRD.

The fact that the domain is not perfectly conserved among the fungi and that the arginine residue is the only invariant aspect may argue that the IRD is not a structural domain required for interaction with other proteins. Rather, it may function as an important element for proper association of the Ras protein with cellular membranes. For example, recent work with human Ras proteins has shown that specific domains within different Ras isotypes affect the way that Ras is oriented with respect to the membrane and, therefore, how well Ras is recognized by effectors for efficient signal transduction. Molecular dynamics modeling employing lipid-bilayer membranes and human Ras isoforms has highlighted important domains of multiple Ras isotypes, including positively charged arginine and lysine residues, that are important for stabilization of membrane orientation when Ras is in its activated conformation (47–49). With these insights, efficient Ras signaling is now understood to involve the structure of the specific Ras isotype in question, the types of Ras posttranslational modifications, and even membrane composition (49). Together, these studies have defined a working model for how different isotypes of Ras proteins in human cells are recognized by different effectors despite the fact that they are so structurally similar. Were the IRD to function in a similar manner for *A. fumigatus* RasA, one would expect multiple downstream effectors be affected. However, our findings show that mutation of the invariant arginine within the IRD to a positively charged lysine is still associated with loss of RasA function. Further exploration concerning the possibility that the fungal IRD may affect Ras-membrane interactions is required.

In summary, we have identified a novel, fungus-specific protein domain that is required for RasA signaling in *A. fumigatus*. The impact of the IRD on Ras signaling in additional pathogenic fungi is of great interest to define its contribution among all fungal organisms. Further exploration could lead to new mechanisms for specifically targeting this highly conserved signaling pathway for antifungal intervention.

## MATERIALS AND METHODS

### Strains, culture conditions, and growth rate analyses.

*Aspergillus fumigatus* strains were maintained on *Aspergillus* minimal medium (AMM), as previously described (50). All strains used in this study are listed in Table 1. For quantification of radial growth rates, 10,000 conidia were point inoculated onto the center of AMM solid agar. The colony diameter of each strain was measured daily over the first 4 days of growth, and images were taken at the end of day 4. Germination experiments were performed as previously described (10). All analyses were performed in triplicate, and data are presented as the mean ± standard deviation.

**TABLE 1:** Strains used in this study

Strain	Genetic background	Source or reference
H237	Wild type	Clinical isolate
Δ*rasA* mutant	H237	Fortwendel et al. (9)
RasAΔIRD	Δ*rasA*	This study
NcAf	Δ*rasA*	This study
ScAf	Δ*rasA*	This study
CnAf	Δ*rasA*	This study
GFP-RasAS3A	Δ*rasA*	This study
GFP-RasAK4A	Δ*rasA*	This study
GFP-RasAF5A	Δ*rasA*	This study
GFP-RasAL6A	Δ*rasA*	This study
GFP-RasAR7A	Δ*rasA*	This study
GFP-RasAR7K	Δ*rasA*	This study
Control strain (GFP-RasA)	Δ*rasA*	Fortwendel et al. (18)
GFP-RasAΔIRD	Δ*rasA*	This study
GFP-RasA/mCh-Cdc42	H237	This study
GFP-RasAΔIRD/mCh-Cdc42	H237	This study

### Construction of mutant strains.

The IRD truncation and point mutations, as well as the N-terminal chimeras, were constructed by incorporating the desired mutation into forward primers used for PCR amplification of previously cloned *rasA* cDNA (Table 2). All primers, both forward and reverse, contained NotI restriction sites to aid in cloning of the newly generated mutant cDNA. Each mutant construct was cloned into the single NotI restriction site in *A. fumigatus* expression vector pAGRP. Vector pAGRP was previously generated to express N-terminally-tagged GFP fusion proteins driven by the *A. fumigatus rasA* promoter (18). Constructs were then screened by PCR and confirmed by sequencing. Confirmed constructs were introduced into the Δ*rasA* mutant using standard protoplasting techniques. Transformants were screened for integration of the construct by PCR and confirmed for expression by Western blot analyses (see Fig. S1 and Fig. S2 in the supplemental material).

**TABLE 2:** Primers used in this study

Primer	Sequence^a^	Use
RasA ΔIRD For NotI	5′-TTTTGCGGCCGCATGGCTGAGTACAAGCTAGTTGTTG-3′	RasAΔIRD mutant
RasA Rev NotI	5′-TTTTGCGGCCGCTTACATAATAACGCATTTTCC-3′	All RasA mutants
ScIRD For NotI	5′-TTTTGCGGCCGCATG**CCACTGAACAAGTCAAACATCCGG**GAGTACAAGCTAGTTGTT-3′	N-terminal chimeras
CnIRD For NotI	5′-TTTTGCGGCCGCATG**TCAAAGGCTCAATTCCTGCGG**GAGTACAAGCTAGTTGTT-3′	N-terminal chimeras
NcIRD For NotI	5′-TTTTGCGGCCGCATG**GCTAACAAGTTCACACGG**GAGTACAAGCTAGTTGTT-3′	N-terminal chimeras
S3A For NotI	5′-TTTTGCGGCCGCATGGCT**GCA**AAGTTCCTTAGAGAGTACAAGC-3′	IRD mutation
K4A For NotI	5′-TTTTGCGGCCGCATGGCTTCA**GCA**TTCCTTAGAGAGTACAAGC-3′	IRD mutation
F5A For NotI	5′-TTTTGCGGCCGCATGGCTTCAAAG**GCA**CTTAGAGAGTACAAGC-3′	IRD mutation
L6A For NotI	5′-TTTTGCGGCCGCATGGCTTCAAAGTTC**GCA**AGAGAGTACAAGC-3′	IRD mutation
R7A For NotI	5′-TTTTGCGGCCGCATGGCTTCAAAGTTCCTT**GCA**GAGTACAAGC-3′	IRD mutation
R7K For NotI	5′-TTTTGCGGCCGCATGGCTTCAAAGTTCCTT**AAA**GAGTACAAGC-3′	IRD mutation
AfCdc42 For NotI	5′-TTTTGCGGCCGCATGGTGGTAGCTACAATT-3′	mCherry-Cdc42 fusion
AfCdc42 Rev NotI	5′-TTTTGCGGCCGCTTACAGCAAGACGCATCT-3′	mCherry-Cdc42 fusion

a Boldface indicates nucleotides changed from the wild-type sequence to introduce the indicated mutation.

The mCherry-Cdc42 chimera was generated by PCR amplification of the *cdc42* coding sequence from *A. fumigatus* cDNA using primers AfCdc42 For NotI and AfCdc42 Rev NotI (Table 2). The *A. fumigatus cdc42* gene (Afu2g05740) was annotated in the Aspergillus Genome Database as the ortholog of the previously characterized *Aspergillus nidulans modA* gene, AN7487 (http://www.aspgd.org). The amplified *cdc42* fragment was then subcloned into the NotI site of vector pUCnCrH. Vector pUCnCrH is a derivative of vector pUCGH (51) and was generated using mCherry gene-specific primers to amplify the mCherry coding sequence as a BamHI/NotI restriction fragment from vector pmCherry-ATG5, a gift from Roberta Gottlieb (Addgene plasmid no. 13095) (52). The mCherry BamHI/NotI restriction fragment was subcloned into pUCGH, replacing GFP, to allow N-terminal fusion of mCherry to a gene of interest cloned into the resulting single NotI restriction site. After *cdc42* subcloning into pUCnCrH, constructs were screened for directional cloning by PCR and sequenced. A confirmed pUCnCrH-Cdc42 construct was finally cotransformed with either the pAGRP-RasA or pAGRP-IRD vector (described above) into the wild-type H237 genetic background using standard protoplasting techniques. Dual selection with hygromycin and phleomycin was performed to select for transformants that successfully integrated both the pUCnCrH-cdc42 and pAGRP-Ras constructs, respectively. Colonies were screened for integration of the fusion constructs by PCR and fluorescence microscopy. Equivalent expression levels of each GFP or mCherry chimera were confirmed by Western blot analysis, as described below.

### Western blot analyses.

*A. fumigatus* strains were grown overnight in AMM plus 0.5% yeast extract at 37°C with shaking at 250 rpm. The resulting hyphal mass was rinsed with sterile, deionized water, and excess water was removed by blotting with filter paper. The hyphal mat was frozen in liquid nitrogen and crushed using a mortar and pestle. The macerated hyphal material was resuspended in extraction buffer (50 mM Tris-HCl [pH 7.5], 150 mM NaCl, 50 mM KCl, 0.01% Triton X-100, 1:100 Pefabloc [Sigma], 1:100 protein inhibitor cocktail [Sigma]), and lysates were centrifuged at 3,500 rpm for 8 to 10 min at 4°C. Supernatants were removed to new tubes, and the protein concentration was quantified using the Bradford method.

Protein samples were diluted 1:1 in Laemmli sample buffer containing 5% β-mercaptoethanol and subjected to SDS-PAGE. Samples were transferred from the gel to polyvinylidene difluoride (PVDF) membrane via a semidry transfer apparatus, and membranes were blocked in 2% nonfat dry milk followed by overnight incubation with primary antibody at room temperature. Membranes were then briefly rinsed in TTBS (Tris-buffered saline with Tween 20) and incubated for 1 h in secondary antibody. For detection, membranes were incubated in SuperSignal West Pico chemiluminescent substrate (Thermo Scientific, Rockford, IL) and visualized using a ChemiDoc XRS system (Bio-Rad, Hercules, CA). The primary antibodies used were the THE GFP antibody (GenScript, Piscataway, NJ) and anti-mCherry antibody 1C51 (Abcam, Inc., Cambridge, MA). The secondary antibodies used were goat anti-mouse IgG2a-horseradish peroxidase (HRP) (for mCherry [Abcam, Inc.]) and goat anti-rabbit IgG-HRP (for GFP [GenScript]). Where densitometry was performed to compare the control and RasAΔIRD strains, band intensity measurements and subsequent analyses were completed using ImageJ software. All experiments were repeated three times.

### RasA activation assay.

Mycelia were obtained by inoculating 5 × 10^7^ conidia in glucose minimal medium with yeast extract (GMM+YE) broth and incubating them at 37°C with shaking at 250 rpm for 20 h. Mycelia were harvested, homogenized under liquid nitrogen, and resuspended in a 1:1 (vol/vol) ratio of lysis buffer containing Mg^2+^ and protease inhibitors (25 mM Tris-HCl [pH 7.5], 20 mM MgCl_2_, 75 mM NaCl, 1 mM EDTA, 1% Igepal CA-630, 2% glycerol, 1:100 Pefabloc [Sigma], 1:100 protein inhibitor cocktail [Sigma]). Total protein lysates were centrifuged at 3,500 rpm at 4°C for 8 min. Supernatants were isolated, and quantification of protein concentration was performed via Bradford protein assay with bovine serum albumin (BSA) as the protein standard.

Pulldown of GTP-bound RasA was accomplished using the Ras activation assay kit (EMD Millipore no. 17-218) following the manufacturer’s protocol, with minor modifications for optimization in the fungal system. Five milligrams of total protein lysate was coincubated with 30 µl of glutathione agarose beads coated with a fusion construct consisting of the Ras binding domain of the human Raf1 protein (Raf1-RBD) fused to a GST tag for 45 min at 4°C with gentle rotation. The Raf1-RBD selectively binds to GTP-bound Ras. Following incubation, the beads were pelleted by centrifugation at 13,000 rpm for 10 s, and the supernatant was discarded. Beads were washed 3 times in 0.5 ml of the lysis buffer described above by centrifugation at 13,000 rpm for 10 s and resuspended in 50 µl lysis buffer. Bound protein was eluted from the beads by boiling and immediately loaded into a 12% SDS-polyacrylamide gel (Bio-Rad). Control samples of 50 µg crude lysate for detection of total Ras levels were similarly boiled and loaded on the gel.

Proteins were transferred to polyvinylidene difluoride (PVDF) membrane (Bio-Rad) and blocked with a solution of 2% skim milk in TTBS for 1 h at ambient temperature. Following blocking, membranes were probed with an anti-Ras, clone RAS10 mouse monoclonal primary antibody (1:2,000 dilution [EMD Millipore]) overnight at 4°C. Membranes were washed 3 times in TTBS for 10 min and incubated with the secondary antibody, a horseradish peroxidase (HRP)-conjugated goat anti-mouse IgG2a (1:2,000 dilution [Abcam, Inc.]), for 1 h. Membranes were washed 2 times in TTBS for 5 min and equilibrated in Tris-buffered saline (TBS) for 10 min prior to detection with the SuperSignal West Pico chemiluminescent substrate (ThermoScientific).

Blots were imaged using the Bio-Rad ChemiDoc XRS HQ System and QuantityOne software (v4.6.5, Bio-Rad). The ratio of GTP-bound RasA to total RasA in each strain was determined by densitometric analysis using ImageJ software. The resulting ratios were then compared using Student’s *t* test (GraphPad Prism v6). The assay was performed in biological triplicates.

### PKA activity assay and coimmunoprecipitation for Ras-Cdc42 interaction.

For measurement of PKA activation, conidia from each strain were inoculated onto 250 ml of either AMM broth (2% glucose) or AMM broth modified to contain 2% glycerol as the sole carbon source. Cultures were incubated at 37°C for 16 h with shaking at 250 rpm followed by either (i) no treatment, or (ii) addition of glucose to a final concentration of 2% and further incubation at 37°C for 2 min to measure response to glucose stimulation. Fungal biomass was then harvested from each culture via vacuum filtration and washed twice with 50 ml sterile water. Whole-cell lysates were generated from harvested fungal biomass, and PKA activity was analyzed using the PepTag nonradioactive cAMP-dependent protein kinase assay (Promega) following the manufacturer’s protocol.

Lysates of each strain were prepared and quantitated as described above. Following the manufacturer’s protocol, equivalent amounts of total protein from each sample were incubated with 30 µl of equilibrated GFP-Trap agarose beads (Allele Biotechnology, San Diego, CA) for 1 h at 4°C, with constant end-over-end rotation. Samples were pelleted by centrifugation at 2,500 × *g* for 2 min, and supernatants were removed. Pelleted beads were then washed three times in 500 µl ice-cold dilution buffer (10 mM Tris-HCl [pH 7.5], 150 mM NaCl, 0.5 mM EDTA, 1:100 Pefabloc [Sigma], 1:100 protein inhibitor cocktail [Sigma]). After washing, the beads and bound proteins were resuspended in Laemmli sample buffer containing 5% β-mercaptoethanol and boiled for 5 min, and the resulting supernatants were subjected to SDS-PAGE. Immunoblotting, imaging, and data analysis were performed described above. Experiments were repeated three times.

### Immunostaining of actin structures and FM4-64 uptake.

Immunostaining of actin structures in *A. fumigatus* wild-type and RasAΔIRD strains was carried out as previously described, with slight modification (53). Sterile glass coverslips were placed into petri dishes containing 20 ml of AMM broth previously inoculated with conidia from each strain. These cultures were then incubated at 37°C for 12 h (control strain) or 16 h (RasAΔIRD), allowing hyphae to form and adhere to the coverslips. After incubation, coverslips with adherent hyphae were removed and washed twice for 5 min in wash buffer [50 mM piperazine-*N*,*N*′-bis(ethanesulfonic acid) (PIPES) at pH 6.7]. Coverslips were next immersed into fixative solution (8% formaldehyde; 50 mM PIPES [pH 6.7], 25 mM EGTA [pH 7.0], 5 mM MgSO_4_, 5% dimethyl sulfoxide [DMSO]) for 30 min at ambient temperature. After fixation, coverslips were washed twice for 10 min in wash buffer to remove excess fixative solution. The fixed hyphae were then inverted onto a 450-µl drop of digestion solution (50 mg/ml VinoTaste [Novozymes], 1.2 M MgSO_4_, 10 mM potassium phosphate buffer [pH 5.8]) for 1 h at ambient temperature. Following digestion, hyphae were washed three times for 5 min each in PE buffer (50 mM PIPES [pH 6.7], 25 mM EGTA) and incubated in methanol at −20°C for 10 min. After methanol incubation, coverslips were washed twice in PE buffer for 10 min each and then incubated at ambient temperature for 1 h with primary antibody (MAB1501 [Millipore] at 1:500 in PE buffer plus 0.5% Igepal CA-630). Hyphae were next washed three times with PE buffer for 10 min each to remove excess primary antibody and then incubated with rhodamine-conjugated secondary antibody (AP124R [Millipore], 1:100 in PE buffer plus 0.5% Igepal CA-630) for 1 h at ambient temperature. Finally, coverslips were washed three times for 10 min each in wash buffer and mounted for microscopy. Images were captured using a 40× objective on a Nikon NiU fluorescence microscope equipped with an mCherry filter cube. Image acquisition exposure times were identical for both strains. The images provided are representative of results achieved from duplicate experiments.

FM4-64 uptake for relative comparisons of endocytosis between the control and RasAΔIRD strains was performed as previously described (31). Briefly, conidia from each strain were inoculated onto AMM broth and incubated in glass-bottom, multiwell plates at 37°C for 12 h (control strain) or 16 h (RasAΔIRD). After this initial incubation period, culture medium was carefully removed from the wells and replaced with AMM containing 10 µM FM4-64 and incubated at 25°C for 2 min. After the 2-min loading incubation, FM4-64-containing medium was carefully removed and replaced with fresh AMM broth. Cultures were then placed in a 37°C environmental chamber mounted on an inverted Nikon Eclipse fluorescence microscope for live cell analysis. Images were captured at the indicated time points, using identical exposure times for each strain. The still frames presented in this article follow the uptake of FM4-64 over time in a single hypha of each strain and are representative of duplicate experiments.
