# Supplementary figures and images for: A Fungus-Specific Protein Domain Is Essential for RasA-Mediated Morphogenetic Signaling in Aspergillus fumigatus
Source: mSphere. 2016 Nov 30;1(6):e00234-16. doi: 10.1128/mSphere.00234-16 (PMC5137380; doi:10.1128/mSphere.00234-16)

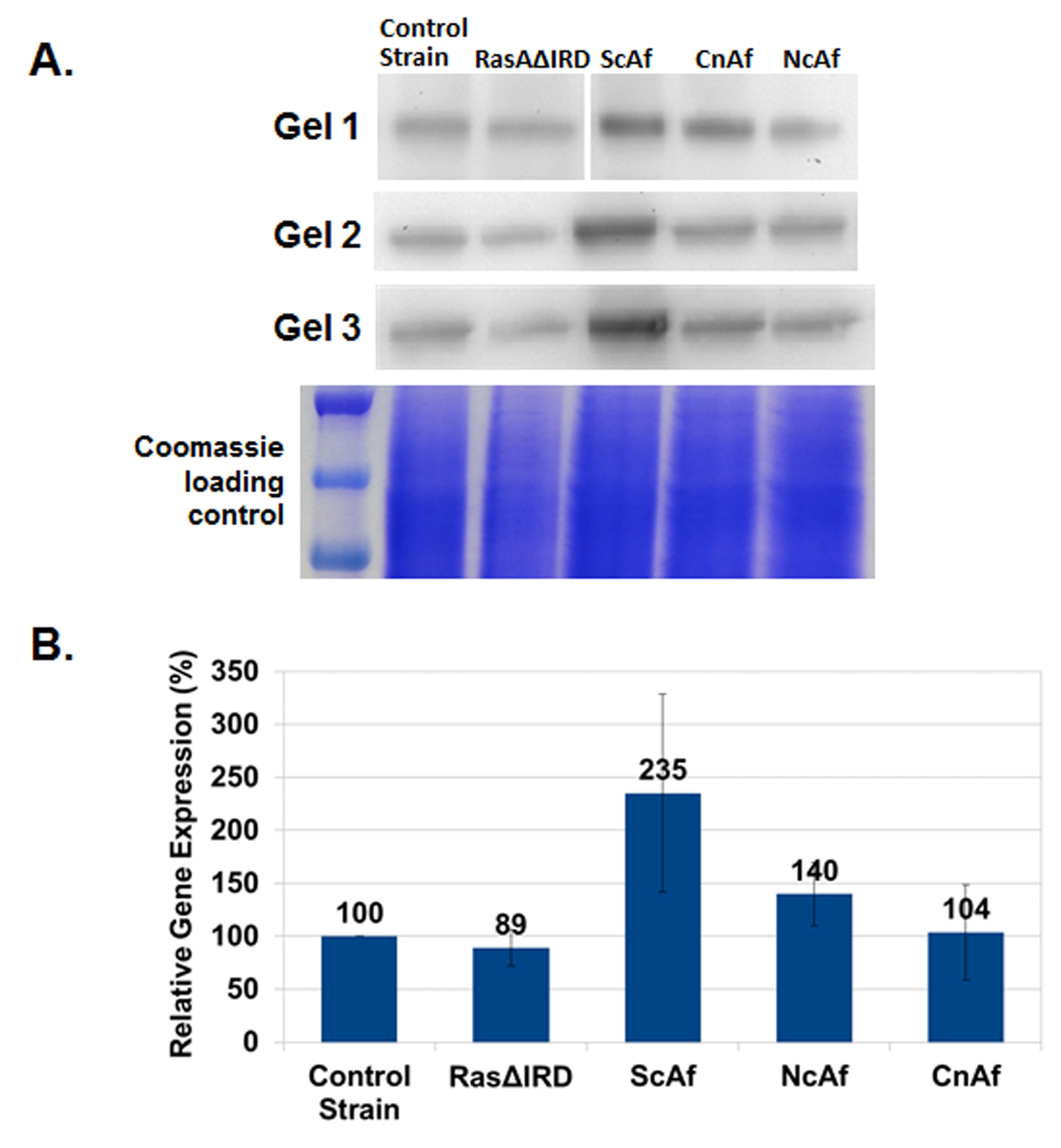

Supplement: Figure S1 [file sph006162199sf1.jpg]

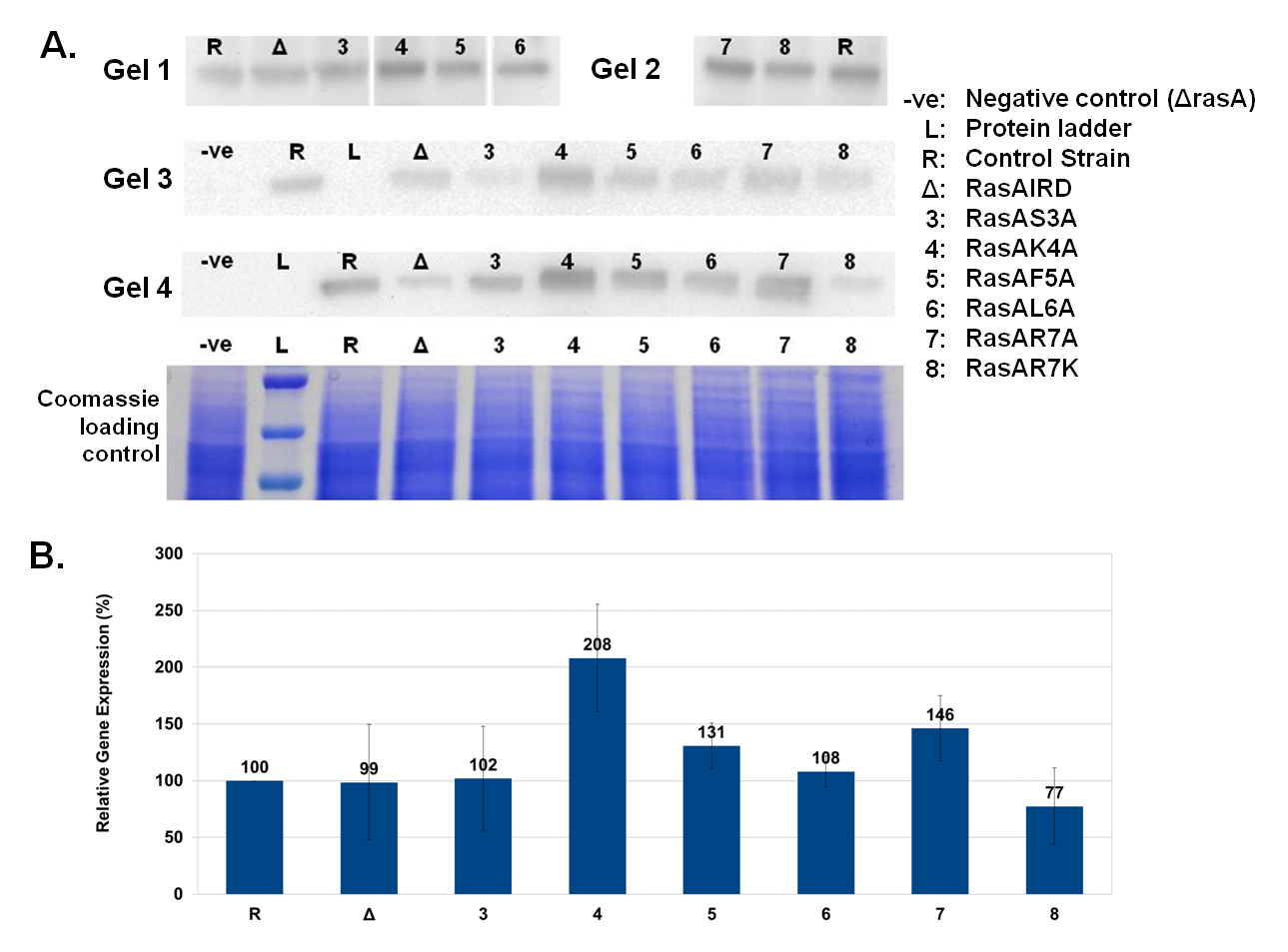

Supplement: Figure S2 [file sph006162199sf2.tif]

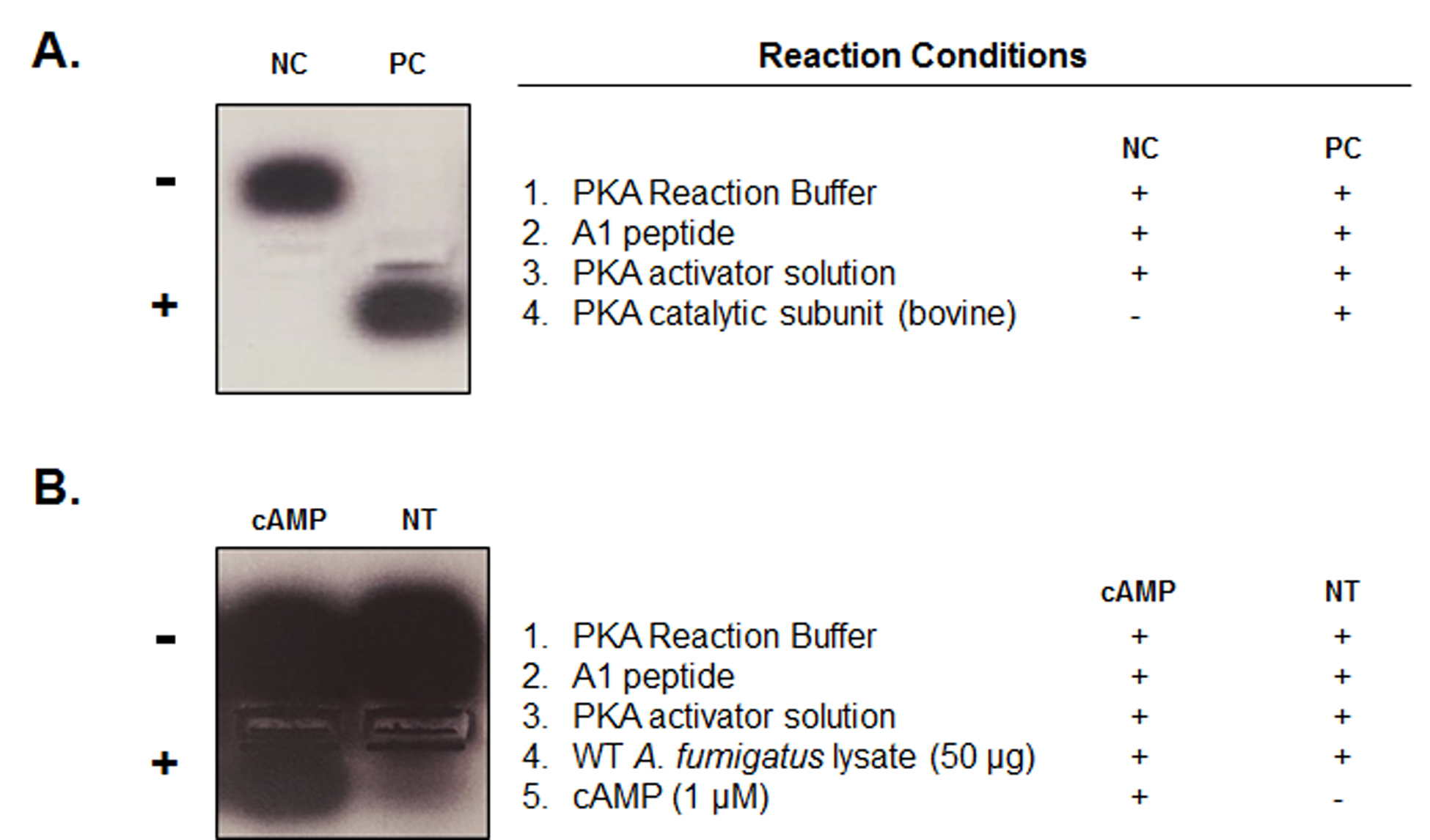

Supplement: Figure S3 [file sph006162199sf3.jpg]

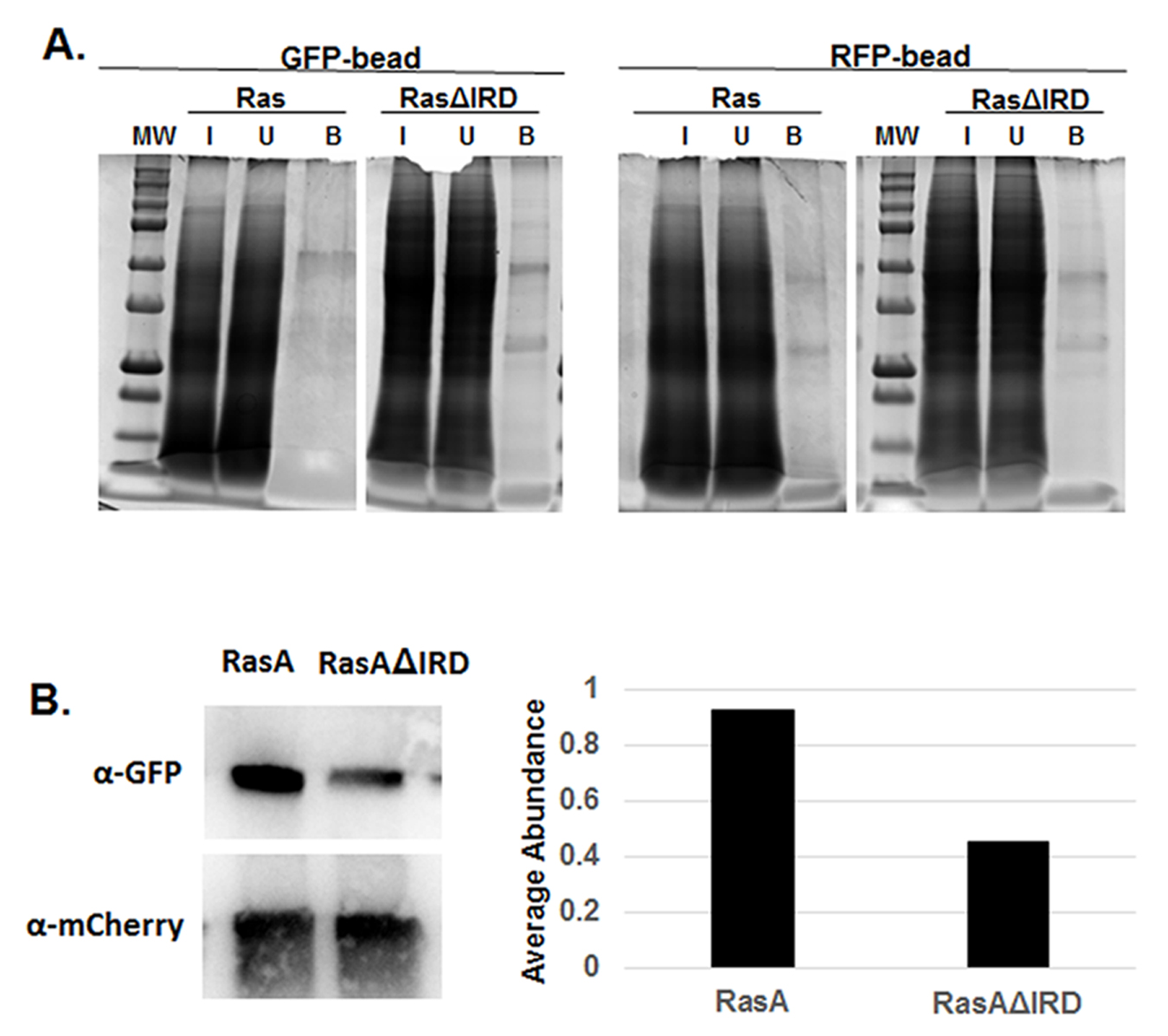

Supplement: Figure S4 [file sph006162199sf4.jpg]
